# Study on Upconversion and Thermal Properties of Tm^3+^/Yb^3+^ Co-Doped La_2_O_3_-Nb_2_O_5_-Ta_2_O_5_ Glasses

**DOI:** 10.3390/ma11081352

**Published:** 2018-08-03

**Authors:** Minghui Zhang, Haiqin Wen, Xiuhong Pan, Jianding Yu, Hui Shao, Fei Ai, Huimei Yu, Meibo Tang, Lijun Gai

**Affiliations:** 1 State Key Laboratory of High Performance Ceramics and Superfine Microstructure, Shanghai Institute of Ceramics, Chinese Academy of Sciences, Shanghai 200050, China; zhangminghui@mail.sic.ac.cn (M.Z.); hqwen@mail.sic.ac.cn (H.W.); xhpan@mail.sic.ac.cn (X.P.); yujianding@mail.sic.ac.cn (J.Y.); mbtang@mail.sic.ac.cn (M.T.); gailijun@mail.sic.ac.cn (L.G.); 2 Laboratory for Materials and Structures, Tokyo Institute of Technology, Midori-ku, Yokohama 226-8503, Japan; 3 School of Materials Science and Engineering, Jiangsu University of Science and Technology, Zhenjiang 212003, China; saihuiwork@163.com; 4 School of Materials Science and Engineering, East China University of Science and Technology, Shanghai 200237, China

**Keywords:** containerless processing, upconversion luminescence, thermal properties, oxide glasses

## Abstract

The effect of Yb^3+^ ions on upconversion luminescence and thermal properties of Tm^3+^/Yb^3+^ co-doped La_2_O_3_-Nb_2_O_5_-Ta_2_O_5_ glasses has been studied. Glass transition temperature is around 740 °C, indicating high thermal stability. The effect of Yb^3+^ ions on the thermal stability is not obvious. Both the glass forming ability and the upconversion luminescence first increase and then decrease with the increase of Yb^3+^ ions. The glasses perform low glass forming ability with ΔT around 55 °C. Blue and red emissions centered around 477, 651, and 706 nm are obtained at the excitation of 976 nm laser. The upconversion luminescence mechanism is energy transfer from Yb^3+^ to Tm^3+^ mixed with two- and three- photon processes. The thermal kinetic Differential Thermal Analysis (DTA)-analysis indicates that the average activation energy first increases and then decreases with the increase of Yb^3+^ ions. This result can be introduced in order to improve upconversion luminescence of glasses by crystallization in the future. Tm^3+^/Yb^3+^ co-doped La_2_O_3_-Nb_2_O_5_-Ta_2_O_5_ glasses with good upconversion and thermal properties show promising applications in solid-state laser, optical temperature sensing.

## 1. Introduction

Upconversion luminescence from near infrared to visible wavelength in rare earth ions doped glasses has attracted much attention for the promising applications in solid-state visible laser [1], optical temperature sensing [2], optical fiber and amplifier [3], and sea water communication [4]. As one typical rare earth ion that can emit blue emissions by upconversion luminescence, Tm^3+^ ion has been paid close attention recently [5,6,7]. Nowadays, 980 nm continuous laser is usually used to be the exciting source, because this laser is commercial and cheap. However, Tm^3+^ ions cannot absorb the wavelength of 980 nm laser directly. Fortunately, Yb^3+^ ion, which is a typical sensitizer in the upconversion luminescence process, shows large absorption efficiency at the wavelength of 980 nm. Yb^3+^ ion can absorb the incident energy and transfer the energy to Tm^3+^ ion effectively. In this way, the Tm^3+^ ion can be excited from ground state to excited levels. So, Yb^3+^ is a good sensitizer for Tm^3+^. Tm^3+^/Yb^3+^ co-doped glasses have been researched fiercely for the blue upconversion luminescence and their promising applications. 

To achieve good upconversion luminescence properties, glass matrix with low phonon energy, high thermal stability, good mechanical properties, and good dissolution for rare earth ions is preferred. Novel Nb_2_O_5_-based glasses perform low phonon energy (~735 cm^−1^), high refractive index, good thermal properties, and high transparency, indicating that it can be a favorable candidate for strong upconversion luminescence from Tm^3+^/Yb^3+^ ion pair [8,9,10]. This new glass is prepared without adding any glass network formers. In this case, traditional crucible experiment technique cannot complete the preparation of this new glass. Therefore, containerless processing method is introduced. This method can constrain heterogeneous nucleation, obtain deep undercooling, and achieve fast solidification. This method is often used to fabricate bulk glasses with low glass forming ability and new meta-stable materials. In the previous study, the containerless processing method is successfully employed to fabricate special glass materials with high performance [11,12,13]. So it can be expected that Tm^3+^/Yb^3+^ co-doped La_2_O_3_-Nb_2_O_5_-Ta_2_O_5_ (LNT) glasses prepared by containerless processing would show good upconversion and thermal properties. 

In this work, the aerodynamic levitation method was used to prepare new Tm^3+^/Yb^3+^ co-doped LNT bulk glasses. To obtain the glass sample with optimal properties, Tm^3+^/Yb^3+^ co-doped LNT glasses with different contents of Yb^3+^ ions were fabricated. As an important practical property, thermal stability was characterized by measuring the Differential Thermal Analysis (DTA) curves. The effect of Yb^3+^ ions on the upconversion luminescence of LNT glasses was studied by fluorescence spectra. Moreover, the emission mechanism was discussed based on the energy level of rare earth ions. DTA curves of glasses with different contents of Yb^3+^ were recorded by different heating rates. The thermal kinetic DTA-analysis was discussed in order to obtain the activation energy (E_a_) by the Kissinger method. In this way, the crystallization process of glasses during the heat treatment can be revealed. This result can be used to be a reference to decide the heat treatment conditions for crystallization to optimize the upconversion luminescence. 

## 2. Experimental

Aerodynamic levitation furnace (self-developed) with heating lasers was introduced to prepare Tm^3+^/Yb^3+^ co-doped LNT glasses with different concentrations of Yb^3+^ ions. The constitutes of Tm^3+^/Yb^3+^ co-doped LNT glasses were 0.65Nb_2_O_5_-(0.29 − *y*)La_2_O_3_-0.01Tm_2_O_3_-*y*Yb_2_O_3_-0.05Ta_2_O_5_ (*y* = 0, 0.03, 0.05). High-purity Nb_2_O_5_ (4N), La_2_O_3_ (4N), Tm_2_O_3_ (4N), Yb_2_O_3_ (4N), and Ta_2_O_5_ (4N) powders were mixed thoroughly in ethanol in stoichiometry composition. The resulted powders were compressed and sintered to obtain dense rod-like samples. Then, the sample was levitated by O_2_ in the aerodynamic levitation furnace and melted by heating laser. After stable levitation, the melt sample was quenched into a glass sphere by containerless solidification. Finally, glass spheres with a diameter of ~3 mm were successfully prepared. The preparation was described in detail in the previous study [8,14,15]. The resulted glass spheres were then polished by two sides to be 1.5 mm thickness wafers for later measurements. 

To study the thermal stability and thermal kinetic analysis, DTA curves of glass spheres were recorded at heating rates of 5, 10, 15, and 20 °C/min in the air by thermal analysis equipment (NETZSCH STA 449C, Selb, Germany). Upconversion luminescence spectra of Tm^3+^/Yb^3+^ co-doped LNT glasses with different concentrations of Yb^3+^ ions were measured at the excitation of 976 nm continuous laser by a spectrofluorometer (Edinburgh instruments FLS980, Edinburgh, UK). The excitation power is set to be 220 mW. To study the upconversion luminescence process, the spectra were tested at different excitation powers of 976 nm laser. Together with energy level structure of rare earth ions, the emission mechanism was discussed.

## 3. Results and Discussion

**Figure 1:**
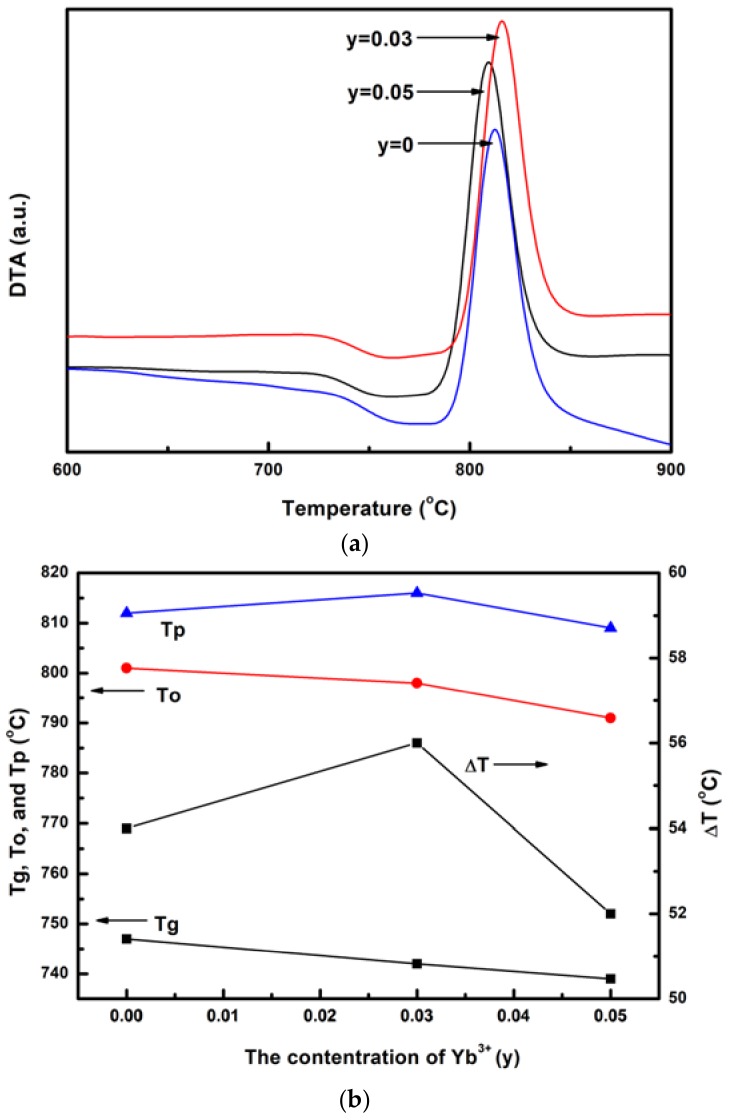
(**a**) Differential Thermal Analysis (DTA) curves of Tm^3+^/Yb^3+^ co-doped La_2_O_3_-Nb_2_O_5_-Ta_2_O_5_ (LNT) glasses with different contents of Yb^3+^ ions; (**b**) The relationship between the values of T_g_, T_o_, T_p_, ΔT, and Yb^3+^ content.

To study the thermal properties of Tm^3+^/Yb^3+^ co-doped LNT glasses, glasses with different contents of Yb^3+^ ions were characterized by DTA. The results are presented in Figure 1. All of the DTA curves have a single glass transition and an exothermic peak ascribed to the crystallization. Based on the curves, we can determine the value of the glass transition temperature T_g_, the onset temperature of crystallization T_o_, and the peak temperature T_p_. After analysis, the values of T_g_ and T_o_ can be evaluated to be around 740 and 800 °C, while typical upconversion fluoride materials ZBLAN glasses show only ~265 °C of T_g_ [16]. So, this glass has high thermal stability, which is favorable for applications. In Figure 1b, the effect of Yb^3+^ ions on T_g_, T_o_, T_p_, and ΔT of glasses is performed. It can be concluded that Yb^3+^ ions show little effect on the values of T_g_, T_o_, and T_p_. This is because La_2_O_3_ is substituted by Yb_2_O_3_ partially. Moreover, the melting point temperatures of La_2_O_3_ and Yb_2_O_3_ are similar. So, the change of T_g_, T_o_, and T_p_ is small with the increase of Yb_2_O_3_. Generally, the difference ΔT between T_o_ and T_g_ is often used to evaluate the glass forming ability. The increase of ΔT often results in the increase of the glass spherical size [17]. From Figure 1b, the value of ΔT first increases and then decreases with the increase of Yb^3+^ content. When *y* = 0.03, the glass forming ability is the largest. When *y* is from 0 to 0.03, Yb_2_O_3_ is added into the sample, which can increase the number of the composition type for the glass. This would be helpful to improve the viscosity of the melt and then increase the glass forming ability. However, when the content of Yb_2_O_3_ is further increased in Tm^3+^/Yb^3+^ co-doped LNT glasses, the glass forming ability is decreased. Totally, Tm^3+^/Yb^3+^ co-doped LNT glasses perform low glass forming ability. The values of ΔT are around 55 °C, which is relatively low and is difficult to form bulk glasses by conventional container methods. So, containerless processing is introduced in order to prepare this new glass. With the advantages in preparing glasses, aerodynamic levitation method is successfully used to fabricate bulk Tm^3+^/Yb^3+^ co-doped LNT glasses. 

**Figure 2:**
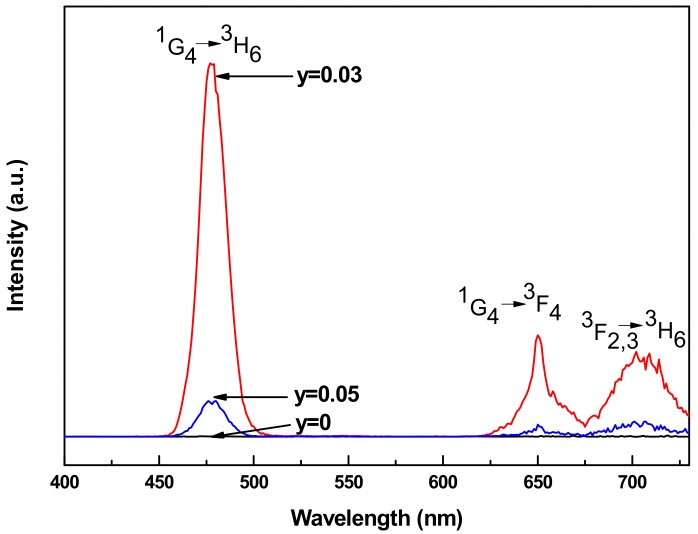
Upconversion luminescence spectra of Tm^3+^/Yb^3+^ co-doped LNT glasses with different Yb^3+^ contents pumped at 976 nm laser.

Upconversion luminescence spectra were recorded at room temperature at the excitation of 976 nm laser. The resulted emission spectra are presented in Figure 2. According to the results, it can be seen that the emission intensity of LNT glasses with *y* = 0 almost cannot be detected. So Tm^3+^ ions cannot absorb the incident pump power of 976 nm laser without the help of sensitizer Yb^3+^ ions. This also indicates that the glass samples are not polluted by other active rare earth ions, which can absorb 976 nm laser. In addition, the spectra of LNT glasses with different Yb^3+^ contents perform similar features, except the changes in intensities of the emission bands as Yb^3+^ doping concentration changes. From Figure 2, blue and red upconversion luminescences are obtained from Tm^3+^/Yb^3+^ co-doped LNT glasses. Three emission bands centered around 477, 651, and 706 nm are observed in the spectra, ascribing to the transitions of ^1^G_4_ → ^3^H_6_, ^1^G_4_ → ^3^F_4_, and ^3^F_2,3_ → ^3^H_6_ in Tm^3+^ ions. Blue emission is much stronger than the red emissions. As the concentration of Yb^3+^ ions increases, the intensity of all the emission bands first increases and then decreases. In the range of low concentration, Yb^3+^ ions can act an excellent sensitizer for Tm^3+^ ions to improve the upconversion luminescence. However, the emission would be quenched if increasing the Yb^3+^ concentration further. In this case, the quenching concentration of Yb^3+^ ions can be determined to be ~0.03 in Tm^3+^/Yb^3+^ co-doped LNT glasses. 

**Figure 3:**
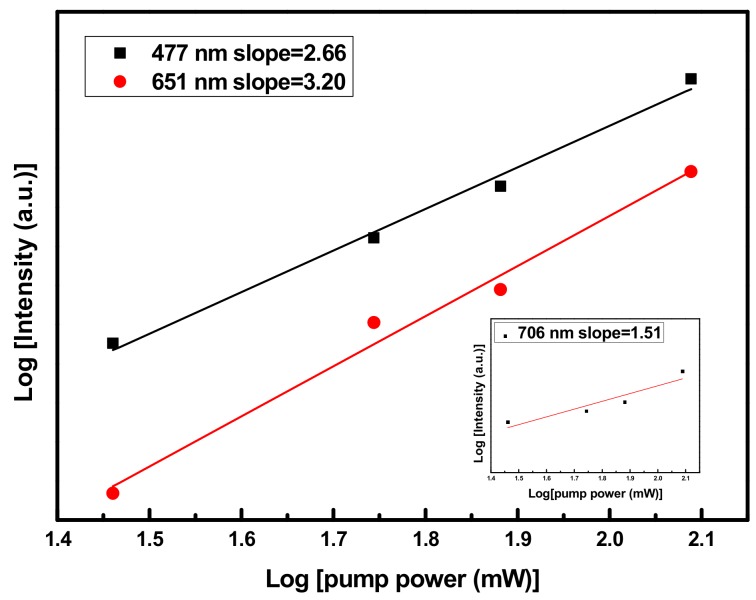
The log-log plot of the emission intensity centered around 477, 651, and 706 nm versus the pump power of Tm^3+^/Yb^3+^ co-doped LNT glasses with *y* = 0.03.

The upconversion luminescence spectra of Tm^3+^/Yb^3+^ co-doped LNT glass with *y* = 0.03 at different pump powers were measured to study the emission process. According to the results, the relationship between the emission intensity *I* and the pump power *P* can be determined. It has been indicated that *I* is propositional to the n-th power of *P* in the form of *I*∞*P*^n^ [18]. Here, n is the number of pump photons that are required to excite the active rare earth ions. Therefore, the relationship between emission intensity *I* and pump power *P* in the logarithm forms is linear. Moreover, the pump photon number n is the slope of the line. The dependency of emission intensity on pump power is plotted in the logarithm forms. The log-log plot is presented in Figure 3. The slopes of blue and red emission bands are 2.66, 3.20, and 1.51, respectively. So, it can be known that the dominant population approach of ^1^G_4_ excited state in Tm^3+^ ions is a three-photon process, while that of the ^3^F_2,3_ excited state is a two-photon process. Tm^3+^ ions can not absorb directly the energy of incident 976 nm laser. Yb^3+^ ions play an important role as a sensitizer in the upconversion luminescence process. The pump power of 976 nm laser is absorbed by Yb^3+^ ions in the glasses. Then, Yb^3+^ ions transfer energy to Tm^3+^ ions by transiting from excited states to ground states. So, the main upconversion luminescence mechanism of Tm^3+^/Yb^3+^ co-doped LNT glasses at 976 nm excitation is Energy Transfer (ET) from Yb^3+^ ions to Tm^3+^ ions. 

**Figure 4:**
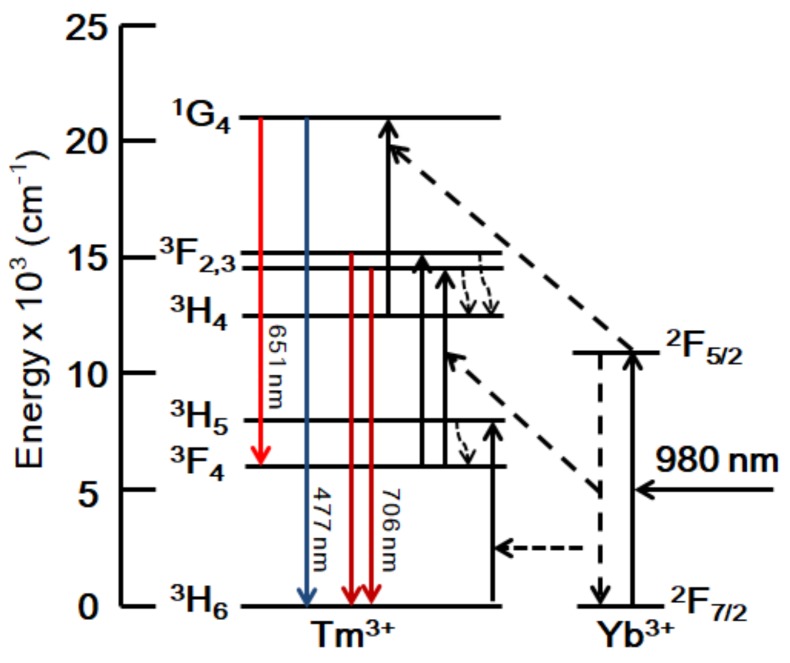
Schematic diagram of upconversion luminescence mechanism in Tm^3+^/Yb^3+^ co-doped LNT glasses excited at 976 nm laser.

According to the energy level structure of rare earth ions, the upconversion luminescence mechanism is discussed in Figure 4. The population of the excited states in Tm^3+^ ions can be revealed. Yb^3+^ ions can efficiently absorb the incident photon and then be excited to ^2^F_5/2_ level from the ^2^F_7/2_ ground state. With the assistance of phonons, Tm^3+^ ions can be excited by energy transfer from Yb^3+^ ions. Tm^3+^ ions in ^3^H_6_ ground state are excited to ^3^H_5_ state by the neighboring excited Yb^3+^ ions. This ET process can be described as ^3^H_6_(Tm^3+^) + ^2^F_5/2_(Yb^3+^) → ^3^H_5_(Tm^3+^) + ^2^F_7/2_(Yb^3+^). Subsequently, Tm^3+^ ions can transit to ^3^F_4_ state by nonradiative relaxation. The same Tm^3+^ ions in ^3^F_4_ states can absorb another photon from neighboring excited Yb^3+^ ion by another ET to be excited to ^3^F_2,3_ states. So, the red emission centered around 706 nm can be generated by the transition from ^3^F_2,3_ to the ground state ^3^H_6_ in Tm^3+^ ions. Moreover, Tm^3+^ ions in ^3^F_2,3_ states can relax to ^3^H_4_ state by nonradiative relaxation. ^3^H_4_ state can be further excited to ^1^G_4_ state by ET process with the help of phonons in Tm^3+^ ions, which can be described as ^3^H_4_ (Tm^3+^) + ^2^F_5/2_(Yb^3+^) → ^1^G_4_(Tm^3+^) + ^2^F_7/2_(Yb^3+^). Blue and red emissions can be emitted by the transitions of ^1^G_4_ → ^3^H_6_ and ^1^G_4_ → ^3^F_4_, respectively. So, the upconversion luminescence mechanism of Tm^3+^ ions in Tm^3+^/Yb^3+^ co-doped LNT glasses is ET mixed with two- and three-photon processes. 

**Figure 5:**
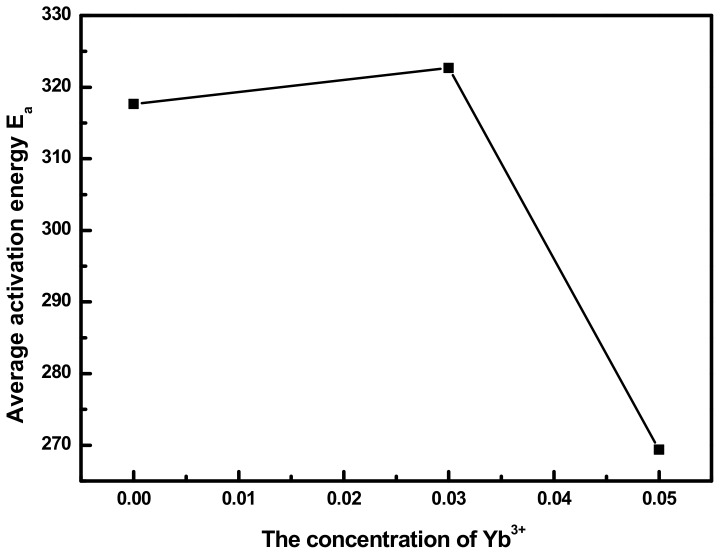
The relationship between the average activation energy and Yb^3+^ concentration in Tm^3+^/Yb^3+^ co-doped LNT glasses.

In our previous study, crystallization was often employed to optimize upconversion luminescence of containerless glasses [19,20]. Crystallization during the heat treatment is very important to improve the emission intensity. The effort would be effective if the crystallization process is controllable. So the crystallization kinetics of glasses should be studied to provide fine heat treatment methods. Furthermore, different compositions of glasses have different kinetic parameters, indicating different optical heat treatment conditions to get strong emissions. Crystallization kinetics would be helpful in determining the heat treatment conditions for different compositions of glasses. In this work, non-isothermal kinetic analyses were introduced. Tm^3+^/Yb^3+^ co-doped LNT glasses were heated at different rates of 5, 10, 15, and 20 °C/min to obtain DTA curves. Then, the Kissinger method was used based on these DTA curves to calculate the activation energy under non-isothermal conditions. The Kissinger equation is written as ln(β/T_p_^2^) = ln[(AR)/E_a_] − E_a_/(RT_p_) [21]. Here, E_a_ is the activation energy. A is the pre-exponential factor. β is the heating rate. The average activation energy of Tm^3+^/Yb^3+^ co-doped LNT glasses with different contents of Yb^3+^ ions can be obtained by the Kissinger method. The result is presented in Figure 5. With the increase of Yb^3+^ ions, the average activation energy first increases and then decreases. Activation energy can be used to evaluate the difficulty of crystallization in glasses. According to the result, the addition of Yb^3+^ ions increase the difficulty of crystallization in Tm^3+^/Yb^3+^ co-doped LNT glasses. However, with the increase of Yb^3+^ ions further, crystallization become easier. Therefore, different compositions of LNT glasses have different kinetic parameters, which need different heat treatment conditions to optimize upconversion luminescence. According to the kinetic result, it is helpful to get suitable crystallization conditions for different compositions of LNT glasses and increase the emission intensity. 

## 4. Conclusions

Tm^3+^/Yb^3+^ co-doped LNT glasses with different Yb^3+^ contents were prepared by the aerodynamic levitation method. The upconversion luminescence and thermal properties of glasses were studied. DTA results indicate that the values of T_g_, T_o_, and T_p_ have little change with the increase of Yb^3+^ ions. Due to the similarity of La_2_O_3_ and Yb_2_O_3_ in melting temperature, Yb_2_O_3_ cannot change the thermal stability of glasses largely. Moreover, the values of T_g_ and T_o_ are evaluated to be around 740 and 800 °C, indicating high thermal stability of glasses. The glass forming ability first increases and then decreases with the increase of Yb^3+^ content. Tm^3+^/Yb^3+^ co-doped LNT glasses perform low glass forming ability with ΔT around 55 °C. Blue and red emissions centered around 477, 651, and 706 nm are observed in upconversion luminescence spectra at the excitation of 976 nm. The emission intensity first increases and then decreases as the Yb^3+^ concentration increases. According to the results of spectra excited at different powers, emissions around 477 and 651 nm are three-photon process, while two-photon process of the emission around 706 nm. The upconversion luminescence mechanism is discussed by ET from Yb^3+^ to Tm^3+^. The thermal kinetic DTA-analysis of Tm^3+^/Yb^3+^ co-doped LNT glasses is studied by DTA curves at different heating rates. The average activation energy, which is calculated by the Kissinger method, first increases and then decreases with the increase of Yb^3+^ ions. This result can be a reference for the heat treatment to improve upconversion luminescence of glasses in the future.
